# Clinical Features and Outcomes Differ between Skeletal and Extraskeletal Osteosarcoma

**DOI:** 10.1155/2014/902620

**Published:** 2014-09-09

**Authors:** Sheila Thampi, Katherine K. Matthay, W. John Boscardin, Robert Goldsby, Steven G. DuBois

**Affiliations:** ^1^Department of Pediatrics, UCSF School of Medicine and UCSF Benioff Children's Hospital, 505 Parnassus Avenue M649, P.O. Box 0106, San Francisco, CA 94143, USA; ^2^Department of Medicine and Epidemiology and Biostatistics, UCSF School of Medicine 505 Parnassus Avenue, San Francisco, CA 94143, USA

## Abstract

*Background*. Extraskeletal osteosarcoma (ESOS) is a rare subtype of osteosarcoma. We investigated patient characteristics, overall survival, and prognostic factors in ESOS. *Methods*. We identified cases of high-grade osteosarcoma with known tissue of origin in the Surveillance, Epidemiology, and End Results database from 1973 to 2009. Demographics were compared using univariate tests. Overall survival was compared with log-rank tests and multivariate analysis using Cox proportional hazards methods. *Results*. 256/4,173 (6%) patients with high-grade osteosarcoma had ESOS. Patients with ESOS were older, were more likely to have an axial tumor and regional lymph node involvement, and were female. Multivariate analysis showed ESOS to be favorable after controlling for stage, age, tumor site, gender, and year of diagnosis [hazard ratio 0.75 (95% CI 0.62 to 0.90); *p* = 0.002]. There was an interaction between age and tissue of origin such that older patients with ESOS had superior outcomes compared to older patients with skeletal osteosarcoma. Adverse prognostic factors in ESOS included metastatic disease, larger tumor size, older age, and axial tumor site. *Conclusion*. Patients with ESOS have distinct clinical features but similar prognostic factors compared to skeletal osteosarcoma. Older patients with ESOS have superior outcomes compared to older patients with skeletal osteosarcoma.

## 1. Introduction

Extraskeletal osteosarcoma (ESOS) is a rare malignant soft tissue sarcoma with histologic similarities to primary bone osteosarcoma but without attachment to the bone or periosteum. ESOS accounts for 1% of all soft tissue sarcomas and 4% of osteogenic osteosarcomas [1]. Multiple case series ranging from 10 to 88 patients have described this rare and unique tumor, with distinct clinical features between ESOS and primary bone osteosarcoma [2, 3].

ESOS is a tumor primarily of older age with a mean age of 47.5 to 61 years [1]. The majority of case series describe a male predominance [1, 3–7]. An early report described an overall survival rate of 38% at 5 years [5]. However, more recent groups that have used multiagent chemotherapy and wide resection during surgical procedures have described overall survival rates of 66 to 77%, similar to skeletal osteosarcoma [7, 8]. Reported adverse prognostic factors include metastatic disease at presentation, large tumor size, and inability to achieve complete surgical resection [1, 4, 5, 7, 9].

In order to further characterize this rare malignancy and compare it statistically to primary skeletal osteosarcoma, we used a large registry to analyze the largest known cohort of patients with ESOS. We define patient and tumor characteristics, estimated overall survival rates, and prognostic factors specifically for patients with ESOS.

## 2. Methods

### 2.1. Patients

In this cohort study, the analytic cohort included patients with osteosarcoma of skeletal or extraskeletal origin reported to the US National Cancer Institute's SEER (Surveillance, Epidemiology, and End Results; http://seer.cancer.gov/data/) system from 1973 to 2009. This registry captures clinical data and outcomes from approximately one-quarter of the US population. Data are collected from a variety of sources, including health providers, pathology reports, laboratories, autopsy reports, and death certificates. Standard data quality measures are employed.

We included a convenience sample of patients in SEER with histologically confirmed high-grade osteosarcoma of any age at time of diagnosis. Given their low metastatic potential, we excluded patients with low-grade osteosarcoma (e.g., parosteal and intraosseous subtypes). The SEER database included 4,178 cases of high-grade osteosarcoma. We excluded five patients with unknown tissue of origin, which resulted in an analytic cohort of the remaining 4,173 patients.

### 2.2. Predictor Variable

We evaluated patient characteristics and outcomes according to tissue of origin (skeletal versus extraskeletal). Primary site was identified in SEER based on ICD-O-3 topography codes, which was then used to code each patient as having a skeletal or extraskeletal primary tumor. As only tumors with the histology code for osteosarcoma were included, primary tumors reported to arise in a soft tissue site were defined as extraskeletal osteosarcoma. Imaging materials (scans and/or scan reports) were not available to confirm skeletal or extraskeletal tissue origin.

### 2.3. Outcome Variables

Patient characteristics and overall survival were evaluated based on extraskeletal versus skeletal disease. Analyzed variables included age at diagnosis (continuous variable and age categorized in tertiles with exclusion of the youngest patients, age ≤ 17 years), sex, year of diagnosis (in 10-year increments), race, primary tumor location (evaluated as distinct sites and also dichotomized with tumors of the head, neck, and trunk defined as axial versus tumors of the extremity defined as appendicular), presence of regional lymph nodes, histologic subtype, tumor size (dichotomized at 10 cm), use of radiation therapy, and stage of disease, defined as either localized or metastatic. Patients with only regional node involvement outside of the primary site of disease were determined to have localized disease.

To determine overall survival, we used total months of follow-up and patient vital status at the time of last follow-up. To determine competing events, we used the variables vital status and cause-specific death, which describes death related to cancer and noncancer causes.

### 2.4. Statistical Methods

We compared patient characteristics between groups with skeletal osteosarcoma or ESOS using chi-squared tests (for categorical variables) or Student's* t*-test (for continuous variables). Overall survival was estimated using Kaplan-Meier (KM) methods with 95% confidence intervals. Potential differences in overall survival between groups were evaluated with log-rank tests. The median follow-up time for the analytic cohort was 102 months.

We also performed competing risk analysis using the Fine-Gray proportional subhazard model to focus on death due to malignancy. Death due to causes other than osteosarcoma was coded as a competing event. If cause of death was unknown, then those patients were not included in the competing risk analysis.

Cox proportional hazard methods were used to determine the effect of extraskeletal disease on overall survival while controlling for other differences in patient characteristics between the groups. Time dependent covariates were used to test the proportional hazards assumption. The SEER database was accessed using SEER∗Stat version 7.1.0.

## 3. Results

### 3.1. Patient Characteristics Differ between Extraskeletal and Skeletal Osteosarcoma

Of the 4,173 patients in the analytic cohort, 256 (6.1%) patients had ESOS.  Table 1 provides patient characteristics according to tissue of origin. The mean age for patients with ESOS was 60.7 years compared to 31.4 years for those with skeletal osteosarcoma (*P* < 0.0001). Patients with extraskeletal involvement were more likely to be female (54.3% versus 44.5%, *P* = 0.002), have an axial tumor (61.1% versus 25.1%, *P* < 0.0001), and have regional lymph node involvement (7.7% versus 2.4%, *P* < 0.0001). There was a statistically significant difference in distribution in primary tumor sites (*P* < 0.0001), most notable in comparing the frequency of primary tumors arising in the lower extremity (32.7% for extraskeletal versus 62.6% for skeletal). Patients with extraskeletal tumors were more likely to receive radiation treatment than patients with skeletal tumors (25.3% versus 12.3%, *P* < 0.0001). Whether radiation was instituted prior to diagnosis of osteosarcoma is unknown. About 30% of ESOS were found in the thorax, which includes involvement of the chest wall, breast, heart, and soft tissue. However, 6 cases were primary lung lesions, 4 were primary pleural lesions, and all are without documented skeletal involvement. Almost all ESOS were reported to have conventional osteosarcoma histology, rather than other histologic subtypes of high-grade osteosarcoma. There were no statistically significant differences in patient race, tumor size, or stage of disease based upon tissue of origin.

### 3.2. Overall Survival Differs between Extraskeletal and Skeletal Osteosarcoma

**Figure 1:**
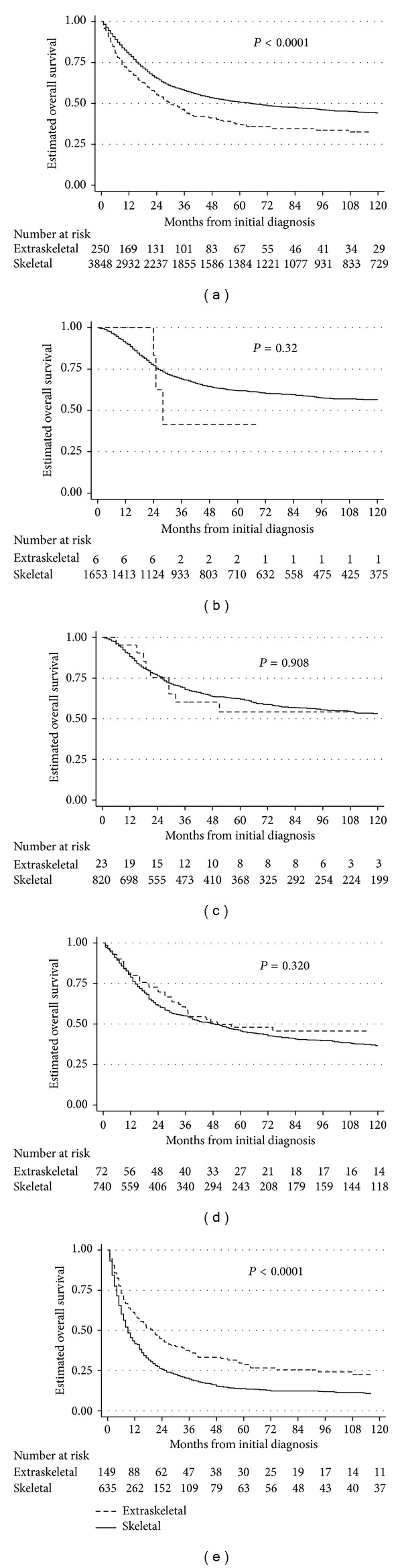
(a) Kaplan-Meier estimates of overall survival from the time of diagnosis according to tumor tissue of origin in all patients with high-grade osteosarcoma [*n* = 4,173 (256 with extraskeletal involvement and 3,917 with skeletal involvement)]. (b) Kaplan-Meier estimates of overall survival from the time of diagnosis according to tumor tissue of origin in patients aged from 0 to 17 years [*n* = 1,672 (6 with extraskeletal involvement and 1,666 with skeletal involvement)]. (c) Kaplan-Meier estimates of overall survival from the time of diagnosis according to tumor tissue of origin in patients aged from 18 to 32 years [*n* = 849 (24 with extraskeletal involvement and 825 with skeletal involvement)]. (d) Kaplan-Meier estimates of overall survival from the time of diagnosis according to tumor tissue of origin in patients aged from 33 to 59 years [*n* = 824 (72 with extraskeletal involvement and 752 with skeletal involvement)]. (e) Kaplan-Meier estimates of overall survival from the time of diagnosis according to tumor tissue of origin in patients aged from 60 to 99 years [*n* = 828 (154 with extraskeletal involvement and 674 with skeletal involvement)].

On Kaplan-Meier testing of the entire analytic cohort, without accounting for competing risks, overall survival was inferior for patients with ESOS as compared to those with skeletal osteosarcoma (Figure 1(a)). The estimated five-year overall survival for those with extraskeletal disease was 37% (95% CI 30.6 to 43.3) compared to 50.8% (95%CI 49.1 to 52.5; *P* < 0.0001) for those with skeletal disease. Differential overall survival was also seen according to tissue of origin when looking exclusively at patients with localized disease, but a statistically significant difference in overall survival according to tissue of origin was not seen in the cohort of patients with metastatic disease (see Supplemental Figure available online at http://dx.doi.org/10.1155/2014/902620). Specifically, the estimated five-year overall survival for those with localized extraskeletal disease was 47% (95% CI 39.8 to 54.6) compared to 63% (95% CI 60.8 to 64.7) for patients with localized skeletal osteosarcoma (*P* < 0.0001). The estimated five-year overall survival for those with metastatic ESOS was 10% (95% CI 2.7 to 23.3) compared to 19% (95% CI 16.4 to 22.4) for those with metastatic skeletal osteosarcoma (*P* = 0.137).

Due to the significantly older age of patients with ESOS, we performed a competing risk analysis to account for death due to other causes in the older population with extraskeletal tumors. Once we controlled for competing events, there was no difference in the cumulative incidence of death from osteosarcoma between patients with extraskeletal or skeletal disease.

Given that the majority of patients with ESOS were > 60 years of age, we compared overall survival of patients with ESOS versus skeletal osteosarcoma categorized by tertiles of age, with the youngest group (age ≤ 17 years) excluded due to too few patients with extraskeletal disease in this group (*n* = 6;Figure 1(b)). We observed that the oldest group of patients with osteosarcoma had inferior overall survival compared to younger age groups (Figures 1(b)–1(e)). However, within this older group, patients with ESOS had superior overall survival compared to patients with skeletal disease (Figure 1(e)). There was no difference in overall survival based on tissue of origin for the remaining two age tertiles (ages 18 to 32 and 33 to 59 years; Figures 1(c)-1(d)).

We constructed Cox proportional hazards models to assess the impact of extraskeletal disease on overall survival independent of potential confounders and again excluding patients ≤ 17 years of age from the analysis. Covariates included in our final model were tertiles of age and sex. Metastatic status, decade of diagnosis, and primary site did not satisfy the proportional hazard assumption; thus we stratified on these covariates in our final model to control for potential differences between groups. After controlling for these variables, extraskeletal disease was predictive of superior overall survival compared to skeletal disease (reference group). The hazard ratio for death for patients with extraskeletal disease was 0.75 (95% CI 0.62 to 0.90; *P* = 0.002) compared to patients with skeletal disease.

To better assess the interaction between age and impact of extraskeletal disease on overall survival, we formally tested for the presence of a statistical interaction by constructing a Cox model of overall survival that included tertiles of age, tissue of origin, and interaction term of these two variables. The *P* value associated with this interaction term was 0.043, confirming a statistically significant interaction between the age and the impact of tissue of origin on overall survival. We next constructed a multivariate Cox model stratified by age tertile in order to obtain tertile-specific estimates of the hazard ratio for death in each tertile according to tissue of origin. As above, this model also controlled for sex, metastatic status, decade of diagnosis, and primary tumor site. We observed that the hazard ratio for death associated with ESOS compared to skeletal osteosarcoma decreased with increasing tertile of age: 1.05 (95% CI 0.51–2.14) for 18–32 years of age; 0.97 (95% CI 0.69–1.37) for 33–59 years of age; and 0.65 (95% CI 0.52–0.82) for 60–99 years of age.

Data on tumor size were not available in 48% of patients. As such, this variable was omitted from the preceding multivariate models but we used this variable to perform a sensitivity analysis that was similar to the final model. Covariates included in this sensitivity analysis were tertiles of age, sex, tumor size, tumor site, and year of diagnosis. Metastatic status did not satisfy the proportional hazard assumption; thus we stratified on this variable. This model yielded a similar estimate of the impact of extraskeletal origin on overall survival compared to our preceding model that did not include tumor size (hazard ratio of 0.79; 95% CI 0.63 to 0.99 for patients with extraskeletal disease compared to patients with skeletal disease; *P* = 0.039).

### 3.3. Prognostic Factors Predictive of Overall Survival in Extraskeletal Osteosarcoma

We next evaluated potential prognostic factors exclusively in the entire cohort of patients with ESOS. On univariate analyses using Cox proportional hazards methods, distant metastatic disease, larger tumor (maximum diameter ≥ 10 cm), older age (age 60 to 99 years), axial tumor site, and regional node involvement were all associated with statistically significantly inferior overall survival (Table 2). Sex and year of diagnosis were not found to be prognostic. We next performed multivariate analysis using Cox proportional hazards models to identify independent adverse prognostic factors in this disease. We included the following significant variables from the above univariate analyses: metastatic status; tumor size; age; and tumor site. Each of these variables remained prognostic on multivariate analysis (Table 2).

We next evaluated potential prognostic factors exclusively in patients with localized ESOS. On univariate analyses using Cox proportional hazards methods, larger tumor size (maximum diameter ≥ 10 cm), axial tumor site, and regional node involvement were all associated with statistically significantly inferior overall survival (Table 3). Age, sex, and year of diagnosis were not found to be prognostic. We next performed multivariate analysis using Cox proportional hazards models to identify independent adverse prognostic factors in patients with localized ESOS. We included the significant variables from the above univariate analyses and both tumor size and axial tumor site remained prognostic on multivariate analysis (Table 3).

Due to limited numbers of patients with data available for both tumor size and regional node involvement, the independent prognostic impact of regional node involvement in this cohort was not assessed as this variable had the fewest patients with available data in our cohort.

## 4. Discussion

In this study of ESOS, we observed that patients with ESOS had significantly different clinical features from patients with skeletal osteosarcoma, including older age, propensity for axial tumors, and female preponderance. Although univariate analysis demonstrated inferior overall survival for patients with extraskeletal disease, once we controlled for competing events, we found that patients with ESOS had a similar cumulative incidence of death due to cancer as patients with skeletal osteosarcoma. Multivariate analysis revealed for the first time that extraskeletal disease was in fact favorable. This effect was driven by an interaction between the age and the impact of tissue of origin on overall survival, with the majority of patients with ESOS being older and with ESOS being favorable in older patients. Adverse prognostic factors previously described for patients with skeletal osteosarcoma were also shown to be prognostic among patients with ESOS.

As reported by other groups, patients with ESOS are significantly older than patients with skeletal osteosarcoma [1, 4–9]. Our cohort included only 6 patients with ESOS < 18 years/1672 (0.4%) patients < 18 years with osteosarcoma, accounting for 2.3% of all patients with ESOS. In contrast to previous reports, we observed a higher incidence of axial primary tumors in patients with ESOS. Regional lymph node involvement in osteosarcoma is a rare and unfavorable finding previously reported by our group [10]. We observed a female predominance for ESOS as did Choi et al., suggesting that gender distribution varies in each cohort due to chance [9]. Understanding the etiology for these clinical differences will likely require greater understanding of the cell of origin of ESOS. The increasing cases of osteosarcoma by year of diagnosis reflect the expanding SEER database in each decade which resulted in more cases being captured and not an increase in the incidence of disease.

Previous literature prior to multimodal therapy reported dismal overall survival for patients with ESOS, while recent groups report similar overall survival with skeletal osteosarcoma [3–9, 11]. Overall survival for our ESOS cohort was inferior but controlling for competing events resulted in similar incidence of death due to cancer. We also observed that the oldest group of patients had the poorest survival, but the presence of extraskeletal disease as compared to skeletal disease renders a more favorable outcome specifically in this age group that accounted for the majority of ESOS cases. This finding could relate to differences in the biology of the tumor with age or differential propensity to perform aggressive surgical resection of soft tissue versus bone tumors in an older population. Unfortunately, neither hypothesis is testable with the data available in SEER. We note that radiation treatment was more likely to be used in extraskeletal tumors, which might suggest a lower rate of aggressive surgical resection of these tumors, though this suggestion cannot be validated in SEER. We also note that although 2% of the patients had osteosarcoma in the setting of Paget's disease, this is not a high enough proportion to account for the difference between groups.

Among patients with ESOS, we found that distant metastatic disease, larger tumor size (≥10 cm), axial tumor site, and older age are adverse prognostic factors. In patients with localized ESOS, axial tumor site and large tumor size were adverse prognostic factors. We confirmed previous reports that distant metastatic disease at diagnosis is an unfavorable prognostic factor in ESOS [1]. Ahmad et al. found that on univariate analysis there was a significant difference in disease specific survival for tumor size >10 cm, microscopically positive surgical margins, and TNM stage >2 [4]. However, none of these factors remained significant in multivariate analysis. In our larger analysis, we were able to confirm the adverse prognostic impact of large tumor size. Lee et al. found on univariate analysis that patients with ESOS with chondroblastic subtype survived longer than those with osteoblastic subtype [6]. Given the lack of histologic heterogeneity of cases of ESOS in SEER, we were not able to evaluate this finding. Goldstein-Jackson et al. found that complete surgical resection was the only statistically significant prognostic factor in their univariate analysis [7]. We note that data on surgical margin and extent of surgical resection were not available for the current analysis, though it seems reasonable to anticipate that these established prognostic factors in osteosarcoma would likewise apply to patients with ESOS.

Use of the SEER database has provided us with a large cohort for this otherwise rare subgroup of osteosarcoma, which has allowed us to analyze potential prognostic factors predictive of overall survival with greater power than other smaller studies. Other strengths with this registry include long-term patient follow-up and diverse population as patients are registered from across the United States. However, use of a registry has limitations, particularly with regard to variables available for analysis. We recognize that tumor size is an important clinical predictor and was only available for 52% of our cohort. We were unable to perform central review of imaging or pathology. In order to obtain a large cohort with ESOS we captured patients over several decades during which treatment has evolved. We were unable to analyze overall survival based on treatment strategies since use of chemotherapy and details on surgical procedure (amputation versus limb sparing or presence of microscopic margins) are either not provided or only sparsely provided in SEER. We also were unable to determine the incidence of each type of recurrence (local versus distant) to confirm previous case series showing both high local and distant recurrent rates [1, 4, 6]. We also did not have access to the percent tumor necrosis after neoadjuvant chemotherapy, which is a known prognostic factor in skeletal osteosarcoma but has not been evaluated in ESOS thus far.

Our study reveals that the incidence of death from ESOS is similar to skeletal osteosarcoma in this cohort of patients and that overall survival varies by age such that the oldest patients have more favorable outcomes with extraskeletal disease as compared to skeletal osteosarcoma. Finally, further prospective research is needed to understand if there are biologic differences between extraskeletal and skeletal osteosarcoma as these differences may explain the variation in patient characteristics and overall survival between the two groups.

## Supplementary Material

The supplemental figure shows the Kaplan-Meier estimates of overall survival from the time of diagnosis according to tumor tissue of origin and stage of osteosarcoma.

## Figures and Tables

**Table 1:** Characteristics of 4,173 patients with osteosarcoma according to tissue of origin.

Characteristic	Skeletal osteosarcoma (*N* = 3,917)	ESOS (*N* = 256)	*P* value
Mean age (range)	31.4 years∗	60.7 years∗∗	<0.0001
	(0–99 years)	(9–96 years)	
Sex			
Male	2,175 (55.5%)	117 (45.7%)	0.002
Female	1,742 (44.5%)	139 (54.3%)	
Year of diagnosis			
1973–1979	395 (10.1%)	9 (3.5%)	<0.0001
1980–1989	609 (15.5%)	21 (8.2%)	
1990–1999	857 (21.9%)	64 (25%)	
2000–2009	2,056 (52.5%)	162 (63.3%)	
Race			
Caucasian	3,031 (77.8%)	212 (82.8%)	0.232
African American	536 (13.8%)	30 (11.7%)	
Asian	297 (7.6%)	12 (4.7%)	
Native American	32 (0.8%)	2 (0.8%)	
Primary site			
Lower extremity	2,392 (62.6%)	80 (32.7%)	<0.0001
Upper extremity	467 (12.2%)	18 (7.3%)	
Head	398 (10.4%)	18 (7.3%)	
Spine	114 (3%)	1 (0.4%)	
Ribs/sternum	105 (2.8%)	0	
Pelvis	343 (9%)	31 (12.7%)	
Thorax	0	70 (28.6%)	
Abdomen/retroperitoneum	0	27 (11%)	
Primary tumor location			
Axial	961 (25.1%)	154 (61.1%)	<0.0001
Appendicular	2,870 (74.9%)	98 (38.9%)	
Regional lymph node			
Present	61 (2.4%)	13 (7.7%)	<0.0001
Absent	2,519 (97.6%)	155 (92.3%)	
Stage			
Distant metastasis	794 (22.3%)	43 (18.6%)	0.188
No distant metastasis	2,763 (77.7%)	188 (81.4%)	
Tumor size			
<10 cm	1,252 (63.1%)	113 (58.9%)	0.241
≥10 cm	731 (36.9%)	79 (41.1%)	
Radiation use			
No	3,374 (87.7%)	186 (74.7%)	<0.0001
Yes	473 (12.3%)	63 (25.3%)	
Histologic type			
Osteosarcoma NOS	3,322 (84.8%)	239 (93.4%)	0.016
Fibroblastic OS	263 (6.7%)	10 (3.9%)	
Telangiectatic OS	128 (3.3%)	3 (1.1%)	
OS in Paget's	79 (2%)	0	
Small cell OS	30 (0.8%)	2 (0.8%)	
Central OS	42 (1.1%)	1 (0.4%)	
Periosteal OS	40 (1%)	0	
High grade surface OS	13 (0.3%)	1 (0.4%)	

OS: osteosarcoma; ESOS: extraskeletal osteosarcoma; NOS: not otherwise specified; totals for each variable may vary due to missing data but percentages reflect the represented data.

∗Median age is 20 years. ∗∗Median age is 64 years.

**Table 2:** Univariate and multivariate prognostic factors for overall survival in a cohort of patients with extraskeletal osteosarcoma.

	Univariate hazard ratio (95% confidence interval)	Univariate *P* value	Multivariate hazard ratio (95% confidence interval)	Multivariate *P* value
Distant metastasis	3.16 (2.11–4.73)	<0.0001	2.39 (1.34–4.25)	0.003
Tumor size ≥10 cm	2.03 (1.40–2.95)	<0.0001	2.11 (1.41–3.17)	<0.0001
Axial tumor	1.98 (1.39–2.80)	<0.0001	1.65 (1.08–2.53)	0.021
Age 18–32 years 33–59 years 60–99 years	Reference 1.30 (0.63–2.70) 2.59 (1.31–5.12)	0.477 0.006	Reference 1.13 (0.45–2.79) 2.53 (1.08–5.90)	0.796 0.032
Regional lymph node present	2.09 (1.14–3.83)	0.017	Not tested	Not tested
Male gender	1.32 (0.96–1.80)	0.086	Not tested	Not tested
Year of diagnosis 1973–1979 1980–1989 1990–1999 2000–2009	1.80 (0.87–3.75) 0.80 (0.44–1.45) 0.85 (0.59–1.23) Reference	0.115 0.465 0.390	Not tested	Not tested

**Table 3:** Univariate and multivariate prognostic factors for overall survival in a cohort of patients with localized extraskeletal osteosarcoma.

	Univariate hazard ratio (95% confidence interval)	Univariate *P* value	Multivariate hazard ratio (95% confidence interval)	Multivariate *P* value
Tumor size ≥10 cm	2.06 (1.36–3.14)	0.001	2.36 (1.53–3.62)	<0.0001
Axial tumor	2.04 (1.35–3.07)	0.001	2.01 (1.30–3.11)	0.002
Age 18–32 years 33–59 years 60–99 years	Reference 1.28 (0.53–3.07) 2.26 (0.98–5.20)	0.580 0.055	Not tested	Not tested
Regional lymph node present	2.33 (1.23–4.41)	0.009	Not tested	Not tested
Male gender	1.27 (0.88–1.85)	0.204	Not tested	Not tested
Year of diagnosis 1973–1979 1980–1989 1990–1999 2000–2009	1.39 (0.49–3.91) 0.69 (0.34–1.42) 0.99 (0.65–1.51) Reference	0.537 0.316 0.964	Not tested	Not tested
