# Supplementary material for: Clinical Features and Outcomes Differ between Skeletal and Extraskeletal Osteosarcoma
Source: Sarcoma. 2014 Sep 9;2014:902620. doi: 10.1155/2014/902620 (PMC4175789; doi:10.1155/2014/902620)
Supplement: Supplementary file 1 — The supplemental figure shows the Kaplan-Meier estimates of overall survival from the time of diagnosis according to tumor tissue of origin and stage of osteosarcoma. [file 902620.f1.doc]

Supplemental Figure


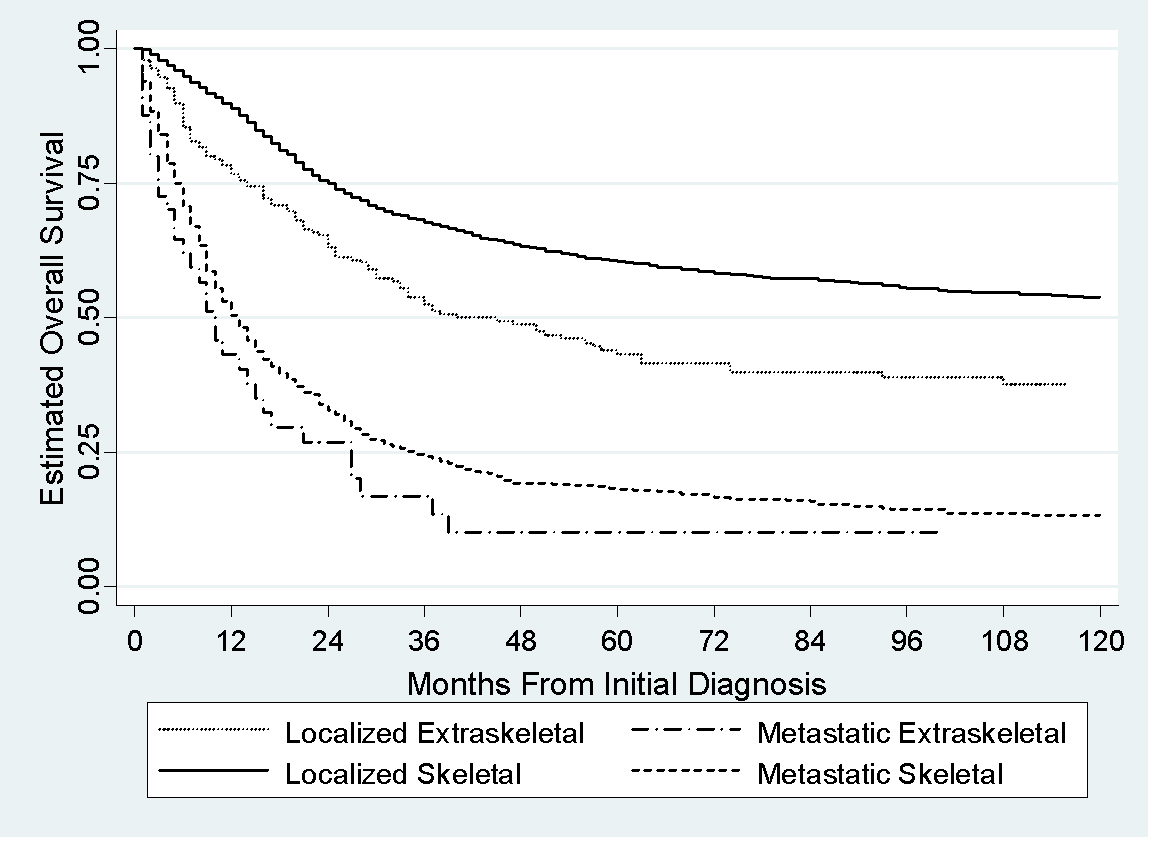


Supplemental Figure: Kaplan-Meier estimates of overall survival from the time of diagnosis according to tumor tissue of origin and stage of osteosarcoma [n= 3,788 (188 with localized extraskeletal tumors, 2,763 with localized skeletal tumors, 43 with metastatic extraskeletal tumors, and 794 with metastatic skeletal tumors)].
